# Regional Incidence of Inflammatory Bowel Disease in a Czech Pediatric Population: 16 Years of Experience (2002–2017)

**DOI:** 10.1097/MPG.0000000000002660

**Published:** 2020-02-12

**Authors:** Petr Jabandziev, Tereza Pinkasova, Lumir Kunovsky, Jan Papez, Martin Jouza, Bara Karlinova, Martina Novackova, Milan Urik, Stefania Aulicka, Ondrej Slaby, Julia Bohosova, Katerina Bajerova, Milan Bajer, Ajay Goel

**Affiliations:** ∗ Department of Pediatrics, University Hospital Brno; † Faculty of Medicine, Masaryk University; ‡ Central European Institute of Technology; § Department of Gastroenterology and Internal Medicine; || Department of Surgery, University Hospital Brno; ¶ Institute of Biostatistics and Analyses Ltd., Brno, Czech Republic; # Department of Pathology, University Hospital Brno; ∗∗ Department of Molecular Diagnostics and Experimental Therapeutics, Beckman Research Institute of City of Hope Comprehensive Cancer Center, Duarte, California, USA.

**Keywords:** children, Crohn disease, Czech Republic, incidence, inflammatory bowel disease, ulcerative colitis

## Abstract

**Objectives:**

Inflammatory bowel disease (IBD) is today a global disease, the incidence of which is growing in the pediatric population. This prospective study aims to decipher IBD incidence and its trend in a pediatric population through 16 years in the South Moravian Region of the Czech Republic.

**Methods:**

We evaluated data concerning 358 pediatric patients with newly diagnosed IBD at University Hospital Brno, which is a gastroenterology center for the entire pediatric population (0–18 years) and cares for all pediatric IBD patients in the South Moravian Region (1,187,667 inhabitants).

**Results:**

The study encompassed 3,488,907 children during 16 years. We diagnosed 192 children (53.6%) with Crohn disease (CD), 123 (34.4%) with ulcerative colitis (UC), and 43 (12.0%) with IBD-unclassified (IBD-U). The incidence of IBD increased from 3.8 (CD 2.9, UC 0.9, and IBD-U 0.0) per 100 000/year in 2002 to 14.7 (CD 9.8, UC 4.0, and IBD-U 0.9) per 100,000/year in 2017 (*P *< 0.001). The overall IBD incidence per 100,000/year was 9.8 (95% confidence interval [CI]: 8.8--10.9). Constituent incidences per 100,000/year were CD 5.2 (95% CI: 4.5--6.0), UC 3.4 (95% CI: 2.8--4.0), and IBD-U 1.2 (95% CI: 0.9--1.6). IBD incidence was projected to reach 18.9 per 100,000/year in 2022.

**Conclusions:**

The overall incidence of pediatric IBD in the Czech Republic is increasing, and especially that of CD, whereas trends in UC and IBD-U appear to be constant. These data highlight the need to identify risk factors involved in the rising incidence of IBD.

**What Is Known**

- The incidence of pediatric-onset inflammatory bowel disease shows geographical variability worldwide.
- The incidence and prevalence of childhood-onset inflammatory bowel disease have risen rapidly in recent decades.

**What Is New**

- This research describes an increase in the incidence of inflammatory bowel disease, mainly because of a rise in the incidence of Crohn disease, between 2002 and 2017 in a population of Czech children.
- Prospectively evaluated incidence rates of pediatric inflammatory bowel disease and its subtypes in the Czech Republic are among the highest in the literature.

Inflammatory bowel disease (IBD), consisting of Crohn disease (CD), ulcerative colitis (UC), and inflammatory bowel disease unclassified (IBD-U), is a chronic relapsing inflammatory disorder with a multifactorial etiology. Both genetic aspects and environmental factors are important for IBD pathogenesis (1,2). Approximately 8% of IBD patients have an early onset of the disease in childhood (2). Reviews in the past decade have concluded that the incidence of pediatric-onset IBD has been rising globally, albeit with great geographic variations (3,4). By analyzing studies of IBD incidence and prevalence from around the world, it has been shown that the incidence is rising not only in high-income, but also in low-income and middle-income countries (5). Nevertheless, that incidence remains much lower in the latter countries (6,7). IBD is associated with higher morbidity and decreased quality of life in patients, results in significantly more frequent use of health care resources, and has become a major health concern (8,9). In the case of children, it is key to realize that the forms of the disease are often more aggressive than in adults, as evidenced by higher rates of immunomodulatory drugs and biologic therapies use compared with adults (10).

We have had relatively limited data on the incidence of IBD in Czech children. Therefore, it would be highly desirable to have more precise data in this field, to describe the exact trends in current developments, and to compare these data with those from other regions, especially within Central Europe.

The aim of our study was to determine the IBD incidence and trends in the Czech pediatric population. The study takes in a 16-year period in the South Moravian Region within the Czech Republic (Fig. 1). It characterizes differences by sex and age, anticipates the incidence of IBD for future years in this region, and, by extension, for the whole of the Czech Republic (11).

**FIGURE 1:**
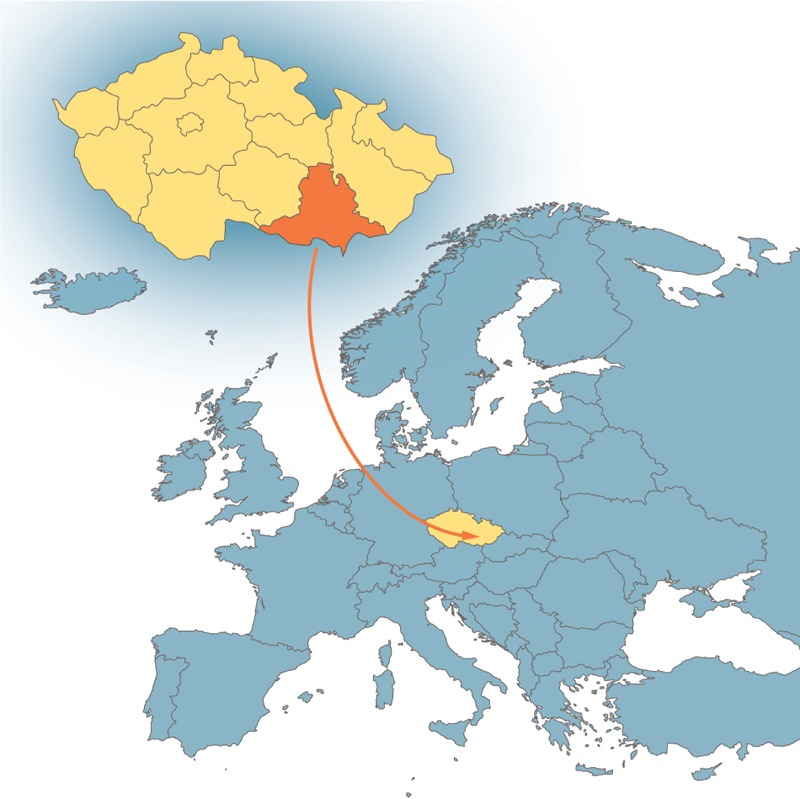
Location of South Moravian Region (orange) within the Czech Republic (yellow) and Europe (gray).

## METHODS

### Study Design

We conducted the study in the South Moravian Region, one of the 14 regional administrative units in the Czech Republic. The region has 1,187,667 inhabitants (2018, Czech statistical office), constituting 11% of the Czech Republic's total population. Children account for 19% of this number, or 229,375. The region covers an area of 7188 km^2^. The health system in the Czech Republic is tax-funded and offers universal access.

The diagnosis and treatment of pediatric patients with IBD in the Czech Republic is limited only to highly specialized centers, which are strategically located and provide comprehensive care to IBD patients. In the South Moravian Region, all regional hospitals are referring pediatric patients for a comprehensive examination to 1 center in the regional capital, University Hospital in Brno. This referral pathway is rigid and has not changed throughout the study period.

Diagnosis of patients under 18 years of age by gastroenterologists serving adults is very unlikely, as in the Czech Republic, this would not be reimbursed by health insurance. The only reason for potential drop-out from our study is diagnosis of a local inhabitant outside of our region. We consider this to be very unlikely, but it does constitute a potential bias in our study. We believe the absolute majority of pediatric IBD patients from the South Moravian region are referred to our center.

The inclusion criteria consisted of children having been diagnosed with IBD according to relevant guidelines (12,13) (clinical history, physical examination, laboratory and serological testing, radiologic findings, and endoscopic appearance with stepwise biopsy for review by clinical pathologists) in a period between January 1, 2002 and December 31, 2017.

All patients underwent upper gastrointestinal endoscopy and ileocolonoscopy, with small bowel imaging (unless typical UC was determined after endoscopy and histology) by magnetic resonance enterography. All children were 0–18 years of age at the time of diagnosis and were resident in the South Moravian Region. The newly diagnosed IBD patients were subdivided into 3 main clinical types: CD, UC, and IBD-U. If a differentiation between UC and CD could not be determined after a complete workup, these patients were designated as IBD-U (11,13). The data were prospectively collected by experienced gastroenterologists into a study database administered by the Institute of Biostatistics and Analyses. Only unequivocal IBD cases were enrolled into the study. The patients without indisputable diagnosis according to the Porto criteria were excluded from further analyses. During the study, data was validated by other experienced gastroenterologists by blindly selecting 10 cases from each year and validating patient records by comparison with original data in the hospital information system. The Institutional Ethical Committee approved the study at University Hospital Brno in accordance with the 1964 Declaration of Helsinki.

### Statistical Methods

Standard statistical methods were adopted for data description. Count data are summarized using absolute and relative frequencies. Median and interquartile range of nonmissing observations are reported for continuous data. Kruskal-Wallis test was used to examine between-group differences for continuous data, whereas Fisher exact test and exact rate ratio test (14), assuming Poisson counts, were applied for count data.

Age-gender adjusted incidence rates are expressed as newly diagnosed children per 100,000 pediatric population per year (100,000/year) and reported with 95% confidence intervals based on a gamma distribution. Data regarding the size of the pediatric population were obtained from the Czech Statistical Office, which counts the number of inhabitants either in actual years or accounts for all regions’ redistributions and adjusts past numbers with regard to the regions’ present sizes. For this study, we chose to work with figures related to actual years. Incidence trends and future projections were estimated using Poisson regression. All statistical significances were evaluated on a level of α = 0.05. The entire analysis was conducted in the statistical software R. Poisson models were estimated using the *glm* function from the built-in *stats* package.

## RESULTS

### Demographics and Inflammatory Bowel Disease Incidence

The basic demographic characteristics of 358 children (<19 years of age) diagnosed for IBD between 2002 and 2017 in the South Moravian Region are shown in Table 1. Over the 16 years, the Czech Statistical Office accounted for 3,488,907 children in the area, and of those diagnosed with IBD, 192 (53.6%) were diagnosed as CD, 123 (34.4%) as UC, and 43 (12.0%) as IBD-U. Among all the IBD cases, 53.1% were boys. The median age of a diagnosed child was 13.9 years (interquartile range: 4.9), the median time between the first symptoms and diagnosis was 4.0 months (interquartile range: 6.3), and no significant differences were found in basic demographic characteristics between diagnoses (all *P* > 0.05).

**FIGURE 2:**
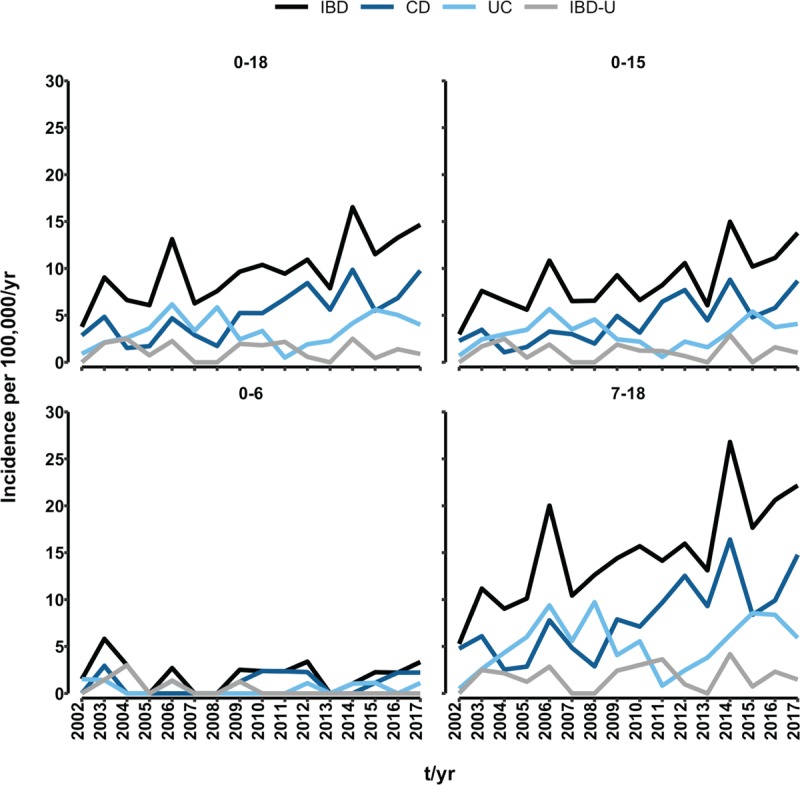
Incidence rates per 100,000/year in different age groups of children (0–18, 0–15, 0–6, 7–18 years of age) newly diagnosed with inflammatory bowel disease (IBD), Crohn disease (CD), ulcerative colitis (UC), and inflammatory bowel disease-unclassified (IBD-U) in the South Moravian Region, 2002 to 2017.

Incidence rates per 100,000/year for the given period 2002 to 2017 are captured in Figure 2. Except for the year 2015, when CD and UC incidences were comparable, the incidence of CD outgrew those of UC and IBD-U after 2009, which had not been the case prior to 2009. Before 2009, no obvious predominance of any diagnosis was observable. A rising trend in IBD incidence is nevertheless noticeable for the entire 16-year period. Although in 2002, the IBD incidence was only 3.8 (CD 2.9, UC 0.9, and IBD-U 0.0) per 100,000/year, in 2017 it reached 14.7 (CD 9.8, UC 4.0, and IBD-U 0.9) per 100,000/year. Although there is no significant difference in CD and IBD-U incidences between years 2002 and 2017 (both *P* > 0.05), this is not the case for IBD (*P* < 0.001) and CD (*P* = 0.003). The predominance of the CD diagnosis after 2009 is confirmed by its being significantly higher than the UC and IBD-U incidences (*P* = 0.029 and *P* < 0.001, respectively) in 2017, but its incidence was only higher than that of IBD-U (*P* = 0.016) in 2002.

A detailed overview of overall incidences by diagnosis, age, and sex is provided in Table 2. The overall IBD incidence per 100,000/year for the 16-year period for children up to 18 years of age was 9.8 (95% CI: 8.8; 10.9), and for children up to 15 years of age, it was 8.6 (95% CI: 7.6--9.8). The overall CD and UC incidences were found to be significantly higher than that of IBD-U (both *P* < 0.001). The difference in CD versus UC incidences also was significant (*P* < 0.001).

### Paris Classification

According to the Paris Classification (15), there were 53 patients (27.6%) in the CD group under 10 years of age (A1a), 115 (59.9%) were between 10 and 17 years of age (A1b), and 28 patients (12.5%) were over 17 years of age (A2). Occurring most frequently was the ileocolic localization (L3) in 134 (69.8%) patients. Upper GIT involvement (L4a and L4b) was found in 28 (14.5%) patients. Most patients (145 [75.5%]), had the nonstricturing and nonpenetrating form of CD (B1), 24 (12.5%) the stricturing form (B2), 16 (8.3%) the penetrating form (B3), and only 1 patient (0.5%) the stricturing and penetrating form (B2, B3). In 6 patients (3.1%) the behavior of the disease could not be validly evaluated. Perianal disease was found in 22 (11.5%) patients. Growth delay at the time of diagnosis was present in 57 (29.7%) CD patients.

Among 123 patients diagnosed with UC, there were 6 (4.9%) patients only with proctitis (E1), 16 (13.0%) with left-sided (distal to splenic flexure) colitis (E2), and 8 (6.5%) with extensive (distal to hepatic flexure) colitis (E3). Pancolitis was present in 90 (73.2%) patients. In 3 patients (2.4%), the extent of the disease could not be validated.

### Inflammatory Bowel Disease by Age

The IBD incidence begins to grow quickly from the age of 8. The peak occurs at 17 years of age, with decrease thereafter. Of those diagnosed with IBD, only 26 (7.3%) were children under 7 years of age, whereas the majority of child patients were at least 7 years of age at diagnosis. Early presentation of CD, UC, and IBD-U at age 6 and earlier occurred in 14 (3.9%), 7 (2.0%), and 5 (1.4%) children, respectively. The overall IBD incidence per 100,000/year for children ages up to 6 years was 2.0 (95% CI: 1.3--3.0), whereas significantly higher (*P* < 0.001) incidence of 14.7 (95% CI: 13.1--16.3) occurred for children aged 7 years and older. Likewise CD, UC, and IBD-U incidences among young children (<7 years) were significantly lower (all *P* < 0.001) than were those for older children (≥ 7 years). No significant between-diagnoses differences were found for patients up to 6 years of age. For patients aged 7 and older, the CD incidence was found to be significantly greater than were those for UC and IBD-U (*P* = 0.001 and *P* < 0.001, respectively), which was true also for incidences of UC vs. IBD-U (*P* < 0.001).

### Inflammatory Bowel Disease by Sex

Of the 358 children diagnosed with IBD, 53.1% were boys (CD 54.2%, UC 52.8%, and IBD-U 48.8%). Overall male and female IBD incidences per 100,000/year were 10.1 (95% CI: 8.7--11.7) and 9.5 (95% CI: 8.1--11.1), respectively, with no statistically significant difference between the 2 (*P* = 0.606). No significant sex-related differences were found between incidences of CD, UC, and IBD-U (all *P* > 0.05). Among both boys and girls, CD and UC incidences were proven to be significantly higher compared with those for IBD-U (all *P* < 0.001) as well as CD vs. UC incidence (*P* = 0.005 and *P* = 0.022 for boys and girls, respectively).

### Trends in Inflammatory Bowel Disease Incidence

**FIGURE 3:**
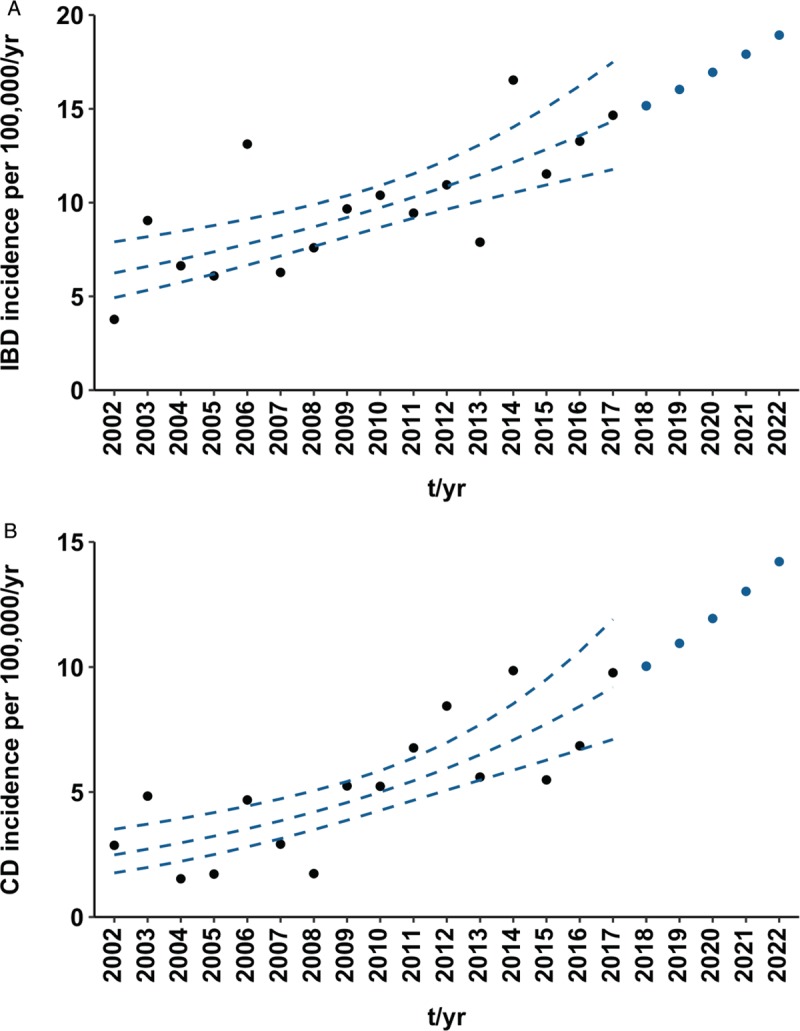
Incidence rates per 100,000/year among children (0–18 years of age) newly diagnosed with inflammatory bowel disease (IBD) (A) and Crohn disease (CD) (B) in South Moravian Region, 2002 to 2017 and 2018 to 2022. Black points represent actual data. Broken blue lines indicate trend over the observed period based on Poisson regression along with a 95% confidence interval. Blue points are future projections.

Figure 3 shows actual IBD and CD incidences per 100,000/year, an estimated trend over the observed 16 years, 95% CI, and future projections for the next 5 years (2018–2022). Overall, the IBD incidence per 100,000/year has risen significantly, with the relative risk (RR) of being diagnosed with IBD increasing by 5.7% each year (RR = 1.057, *P* < 0.001). The rising trend in IBD mainly reflects significant increase in newly diagnosed CD cases (RR = 1.091, *P* < 0.001), whereas neither UC (RR = 1.030, *P* = 0.133) nor IBD-U incidence (RR = 0.988, *P* = 0.723) showed any significant changes. The IBD incidence is projected to reach 18.9 per 100,000/year in 2022 (14.2 for CD).

The relative risk per year of being diagnosed with IBD was 2.7% greater for girls (RR = 1.072, *P* < 0.001) than for boys (RR = 1.045, *P* = 0.005). The difference is substantially smaller for the CD diagnosis, with girls facing relative risk of being diagnosed that is 1.2% higher than in the case of boys (RR = 1.088 [*P* < 0.001], RR = 1.100 [*P* < 0.001] for boys and girls, respectively). There appeared a significant increase in incidence over the 16-year period of girls being diagnosed with UC (RR = 1.065, *P* = 0.026).

## DISCUSSION

We provide a detailed longitudinal data set describing IBD incidence and its trends in pediatric patients through the 16 years between 2002 and 2017 within a well-defined geographical area of the Czech Republic. To our knowledge, this is the most recent and comprehensive study in this field. Our results provide important insights into the high incidence of IBD and its increasing trend, which are due mainly to the rise in rates of CD. The overall IBD incidence per 100,000/year shows IBD incidence in children within the Czech Republic currently to be among the highest in the world (3–6,16,17). These data show considerably higher incidences than were determined from the first Czech national survey conducted about 2 decades earlier by Pozler et al (18). In another, smaller Czech study from the Olomouc Region, an overall increase in the incidence of IBD was also confirmed (19). Schwarz et al recently published a 16-year prospective study of pediatric IBD patients in the Pilsen Region of the Czech Republic showing that a group of 170 pediatric patients (study period 2000–2015) represented an average incidence of IBD per 100,000/year of 10.0 (6.2 for CD, 2.8 for UC, and 1.0 for IBD-U). That study also projected increasing future incidence (11). Our study is based on data from a region of almost similar size but with more than 2 times the number of pediatric patients with IBD. The incidence of IBD subtypes revealed by our group, and including the proportions among them, is practically identical to the results from the Pilsen Region. Moreover, the incidence trends are very similar. Although acknowledging certain limitations, we therefore, can presume that our findings are potentially similar to incidences to be found in pediatric IBD patients throughout the Czech Republic. We clearly demonstrate an overall increase in IBD incidence within the population of Czech children, with the overall incidence rising more than 3 times when comparing data from the first and final year in our data set. The overall IBD incidence per 100,000/year rose significantly over the study period, that trend reflecting mainly the statistically significant increase in CD incidence even as the UC and IBD-U incidences showed no statistically significant changes. Our future projections put the IBD incidence at 18.9 per 100,000/year in 2022 (14.2 per 100,000/year for CD). In the future, we will be able to compare these projections with real data obtained from our patients. In comparison to neighboring countries in Central and Eastern Europe, our data suggests that the increase in the incidence of IBD is particularly noticeable in Hungary (20,21) and Slovenia (22) whereas it seems to be stable in Germany (23). In Austria, on the other hand, an overall increase was observed from 1997 to 2007 in both CD and UC, primarily in the largest urban areas (24). The overall incidence of IBD cases was surprisingly very low in Poland, at 2.7 per 100,000/year (0.6 for CD, 1.3 for UC, and 0.8 for IBD-U) (6,25). No current data on the incidence of IBD in neighboring Slovakia is known at this time. Globally, CD predominates over UC and IBD-U in areas having high IBD incidence. Recent data indicate higher rates of pediatric CD than UC in Europe and North America, except in northern California (26), Finland (27), Poland (25), and Italy (28), where the incidence of UC exceeds that of CD (6). The reasons for these notable differences remain uncertain (4,6,29). In a recent systematic review, Sykora et al analyzed 140 pediatric incidence studies. They demonstrated substantial increase in the incidence of pediatric IBD as well as great geographic variation. The incidence of IBD remains highest in the northern populations of Europe and America but has remained stable or even decreased. Rising rates of pediatric IBD have been observed in previously low-incidence areas and much of the developing world, as well as among children of immigrants. The incidence rates of CD and UC vary worldwide between 0.2/100,000 and 13.9/100,000 and between 0.1/100,000 and 15/100,000, respectively. In time-trend analyses, 67% of CD and 46% of UC studies have reported significant increases (6). Variation in IBD incidence may reflect differences in the distribution of various environmental triggers for a given disease in specific areas. Rapidly changing IBD incidence in such areas create an opportunity for future studies of genetic-environmental interactions (6,30,31). Exposure to environmental factors in childhood appears to be essential for the later development of IBD. In rapidly developing areas, such as Asia, the food composition of traditional human diets is changing. People are shifting from homemade to processed foods, and lifestyles are changing also in other ways. All this may affect the composition of the human intestinal microbiota and potentially be related to increasing IBD (31–35). We can hypothesize that this reflects a certain similarity to the significant rise in the socioeconomic level within the Czech Republic after the close of the communist era, and thus, an influence on the increase in IBD. One of the prerequisites for developing a proper understanding of IBD's pathogenetic context and next steps in better care for pediatric patients is to find accurate and relevant data, including data for IBD's incidence and prevalence in these patients (36).

## Figures and Tables

**TABLE 1:** Demographics of children (0–18 years of age) newly diagnosed with inflammatory bowel disease, Crohn disease, ulcerative colitis, and inflammatory bowel disease-unclassified in the South Moravian Region, 2002 to 2017

Parameter	IBD	CD	UC	IBD-U
Sex, N (%)				
N	358	192	123	43
Male	190 (53.1%)	104 (54.2%)	65 (52.8%)	21 (48.8%)
Female	168 (46.9%)	88 (45.8%)	58 (47.2%)	22 (51.2%)
Age at diagnosis (years)				
N	358	192	123	43
Median (interquartile range)	13.9 (4.9)	14.4 (4.7)	13.6 (4.3)	12.4 (6.7)
Time from first symptoms to diagnosis (months)				
N	301^*^	156	112	33
Median (interquartile range)	4.0 (6.3)	4.0 (6.6)	3.0 (6.2)	5.0 (6.0)

CD = Crohn disease; IBD = inflammatory bowel disease; IBD-U = inflammatory bowel disease-unclassified; UC = ulcerative colitis.

^*^Discrepancy between number of patients and total N is because of missing data.

**TABLE 2:** Inflammatory bowel disease incidence by age and sex in newly diagnosed children (0–18 years of age) with inflammatory bowel disease, Crohn disease, ulcerative colitis, and inflammatory bowel disease-unclassified in the South Moravian Region, 2002 to 2017

Incidence per 100,000/year	IBD	CD	UC	IBD-U
Age				
0–18				
N	358	192	123	43
Mean (95% CI)	9.8 (8.8--10,9)	5.2 (4.5--6.0)	3.4 (2.8--4.0)	1.2 (0.9--1.6)
0–15				
N	257	135	92	30
Mean (95% CI)	8.6 (7.6--9.8)	4.5 (3.8--5.4)	3.1 (2.5--3.8)	1.1 (0.7--1.5)
0–6				
N	26	14	7	5
Mean (95% CI)	2.0 (1.3--3.0)	1.1 (0.6--1.8)	0.5 (0.2--1.1)	0.4 (0.1--0.9)
7–18				
N	332	178	116	38
Mean (95% CI)	14.7 (13.1--16.3)	7.8 (6.6--9.0)	5.2 (4.3--6.2)	1.7 (1.2--2.3)
Gender				
Male				
N	190	104	65	21
Mean (95% CI)	10.1 (8.7--11.7)	5.5 (4.4--6.7)	3.4 (2.6--4.4)	1.2 (0.7--1.8)
Female				
N	168	88	58	22
Mean (95% CI)	9.5 (8.1--11.1)	4.9 (3.9--6.1)	3.3 (2.5--4.3)	1.3 (0.8--1.9)

CD = Crohn disease; CI = confidence interval; IBD = inflammatory bowel disease; IBD-U = inflammatory bowel disease-unclassified; UC = ulcerative colitis.
